# Increasing the reliability of data analysis of functional magnetic resonance imaging by applying a new blockwise permutation method

**DOI:** 10.3389/fninf.2014.00072

**Published:** 2014-08-13

**Authors:** Daniela Adolf, Snezhana Weston, Sebastian Baecke, Michael Luchtmann, Johannes Bernarding, Siegfried Kropf

**Affiliations:** ^1^Institute for Biometry and Medical Informatics, Otto von Guericke University MagdeburgMagdeburg, Germany; ^2^Department of Neurosurgery, Otto von Guericke University MagdeburgMagdeburg, Germany

**Keywords:** SPM analysis, functional MRI, familywise error rate, blockwise permutation including a random shift, autocorrelation

## Abstract

A recent paper by Eklund et al. (2012) showed that up to 70% false positive results may occur when analyzing functional magnetic resonance imaging (fMRI) data using the statistical parametric mapping (SPM) software, which may mainly be caused by insufficient compensation for the temporal correlation between successive scans. Here, we show that a blockwise permutation method can be an effective alternative to the standard correction method for the correlated residuals in the general linear model, assuming an AR(1)-model as used in SPM for analyzing fMRI data. The blockwise permutation approach including a random shift developed by our group (Adolf et al., 2011) accounts for the temporal correlation structure of the data without having to provide a specific definition of the underlying autocorrelation model. 1465 publicly accessible resting-state data sets were re-analyzed, and the results were compared with those of Eklund et al. (2012). It was found that with the new permutation method the nominal familywise error rate for the detection of activated voxels could be maintained approximately under even the most critical conditions in which Eklund et al. found the largest deviations from the nominal error level. Thus, the method presented here can serve as a tool to ameliorate the quality and reliability of fMRI data analyses.

## 1. Introduction

Even after many years of sophisticated fMRI analyses some debate remains about the validity of models based on the general linear model approach (GLM). A main challenge for linear model estimations used in standard software is that the assumption of independent residuals, which is necessary for classical general linear modeling, usually does not hold for fMRI data. Due to the experimental setup and the underlying physiological processes, a temporal autocorrelation exists between successive measurements. This may mainly result from missing signal components in the model as well as from the extended time course of the so-called hemodynamic response function (HRF): even for very short stimuli the HRF usually rises during the first 6–8 s, declines slowly thereafter, and, after a so-called undershoot, returns to the baseline. The time course thus extends over about 25 s, while the intermeasurement interval is usually much shorter. Thus, an interstimulus interval of typically 2 s will lead to overlapping signals and temporal correlations. Other factors causing this correlation may be technical properties of the scanners (changes in the magnetic field, see Smith et al., 1999) or influences of breathing and pulse or other artifacts. Neglecting this autocorrelation leads to high false positive rates. For that reason, an optional AR(1) autocorrelation model is implemented in SPM (Friston et al., 2007), one of the most commonly used software tools for fMRI analyses. Under the assumption that the temporal correlation can be modeled by an AR(1) process, the correlation structure is estimated using a restricted maximum likelihood approach (ReML; Ashburner et al., 2013, Section 8.9) and then plugged into the transformation matrix for prewhitening (Friston et al., 2007).

Furthermore, statistical inference is often performed at the voxel level, which leads to an enormous number of univariate tests that in turn have to be adjusted for multiple testing. In many fMRI analysis software packages, including SPM, the user can choose the level of significance as well as adjust the *p*-values (false discovery rate [FDR]; familywise error rate [FWE]; or no correction). The most conservative choice consists in choosing FWE as an adjustment for multiple testing, typically with a default level of significance of 0.05. Although the FWE option is common (Focke et al., 2008; Baecke et al., 2009; Kahnt and Tobler, 2013), inference is also performed using uncorrected *p*-values (Morcom and Friston, 2012; Causse et al., 2013; Groeschel et al., 2013), especially when applying group analyses. The concept of FDR (Benjamini and Hochberg, 1995; Genovese et al., 2002) constitutes a compromise between the rigid control of FWE and the unadjusted approach (Sorg et al., 2007; Meda et al., 2009; Ahmadi et al., 2013).

However, it is well known that false positives may still result even when applying FWE-corrected analyses, thereby severely restricting the validity of a study. In a recently published empirical study, Eklund et al. (2012) strikingly demonstrated this effect by analyzing resting-state data sets using artificial stimulus protocols. These resting-state data sets had been made publicly available by different groups to give other researchers access to large data sets for replication purposes or in order to develop alternative data analysis approaches.

In resting-state measurements, data are acquired without explicit external stimulation. Even without any specific stimulus, however, there is always some neuronal activity in the so-called default network (Fair et al., 2009). In order to extract inherent information about this ongoing neuronal activity, special data analysis techniques such as independent component analysis and correlation analyses have been used (Biswal et al., 2010; Di et al., 2013). However, applying fixed paradigms such as a block-based stimulus protocol with a deliberately fixed block length should not lead to any statistically activated voxel, given that neuronal activity in the resting state is assumed to float freely between different internal networks.

To test the reliability of the standard approach using a GLM and an AR(1) model as the main option in SPM, Eklund et al. therefore simulated various artificial paradigms and tested whether a change in the BOLD signal response was significantly associated with these paradigms thus leading to spuriously activated regions. If the FWE (nominal test level 0.05) is maintained, then a spuriously significant neuronal activity would be expected in approximately 5% of the data sets (confidence bounds depend on the number of data sets). Even with the FWE adjustment, however, Eklund et al. found significantly activated voxels in up to 70% of the data sets. The authors concluded that the main reason for these inacceptably high familywise error rates appeared to be that the global AR(1) autocorrelation correction in SPM failed to model the spectra of the residuals appropriately. Also other papers report on high rates of false positive results in parametric statistical approaches in neuroimaging. Silver et al. (2011) found a poor control of rejection rates in cluster-size based analyses under both stationary and non-stationary assumptions in an imaging genetics study using voxel-based morphometry. Scarpazza et al. (2013) detected increased rejection rates—also in voxel-based morphometry—when comparing a single individual with a control group.

Against this background, mainly the paper by Eklund et al. motivated us to re-analyze the same resting-state data using an alternative method to adjust for temporal correlation, one that was previously presented by Adolf et al. (2011). There it was shown that a permutation-based approach could approximately meet the nominal familywise test level, leading to more reliable test results. Our method is based on a blockwise permutation strategy that takes into account the correlation between measurements acquired within short time windows by permuting whole blocks of adjacent elements rather than single elements. In this approach, all that needs to be ensured is that the blocks are approximately exchangeable under the null hypothesis. We implemented our method in MATLAB, including SPM8 components, in order to achieve maximal comparability with (Eklund et al.).

In the following section the main principles of the blockwise permutation and our modification are reviewed briefly. Then, we describe the design of the present study in comparison to that of Eklund et al. (2012) and also the implementation of our procedure. The results and the discussion are presented in the subsequent sections.

## 2. Materials and methods

### 2.1. Background and motivation for the blockwise permutation approach

For the univariate GLM, which is applied by the majority of fMRI users, inference can be accomplished voxelwise as well as at the cluster level.

Usually data are first preprocessed. This includes motion correction, optional slice-timing correction, spatial smoothing, global normalization, and high-pass filtering (the latter optionally combined with prewhitening). Prewhitening is the key strategy for taking into account the temporal correlation of the data due. When choosing prewhitening in SPM, an AR(1)-model for each time series (i.e., for every voxel) is assumed and the correlation structure is estimated in a ReML approach as already noted above (Bullmore et al., 2001; Friston et al., 2007). This so-called whitened series of scans is then regarded as uncorrelated and the GLM analysis proceeds as usual.

The results can be adjusted for multiple testing in order to maintain the nominal familywise error rate. Depending on the spatial correlation of the data, a Bonferroni correction or an adjustment according to Gaussian random field theory is made (Friston et al., 2007). Depending on the choice of adjustment (FWE or none), SPM8 determines a threshold for the voxelwise *t*-values and displays the significant voxels as overlays to brain maps.

Eklund et al. (2012) based their analyses on the standard SPM technique of a classical GLM after prewhitening, which is probably the most common approach in the field of fMRI. Additionally, they applied a conventional permutation approach after prewhitening via an AR(4) model. They then analyzed 1482 of 1484 publicly available resting-state data sets (two had to be excluded because of empty brain masks). These data sets can be found in the Neuroimaging Informatics Tools and Resources Clearinghouse data base (NITRC). Data are fully anonymized and access is unrestricted for noncommercial use. For details see http://fcon_1000.projects.nitrc.org. Table 1 provides a basic summary of the data sets.

**Table 1:** **Summary of the resting-state data sets used by Eklund et al. (2012)**.

**Institution**	**Persons**	**No. of subjects**	**TR (s)**	**No. of Time points**	**Volume resolution**
Ann Arbor	Monk, C.S., Seidler, R.D., Peltier, S.J.	25	1.0	295	64 × 64 × 40
Ann Arbor	Monk, C.S., Seidler, R.D., Peltier, S.J.	36	1.0	395	64 × 64 × 16
Atlanta	Mayberg, H.S.	28	2.0	205	64 × 64 × 20
Baltimore	Pekar, J.J., Mostofsky, S.H.	23	2.5	123	96 × 96 × 47
Bangor	Colcombe, S.	20	2.0	265	80 × 80 × 34
Beijing	Zang, Y.F.	198	2.0	225	64 × 64 × 33
Berlin	Margulies, D.	26	2.3	195	64 × 64 × 34
Cambridge	Buckner, R.L.	198	3.0	119	72 × 72 × 47
Cleveland	Lowe, M.J.	31	2.8	127	128 × 128 × 31
ICBM	Evans, A.C.	86	2.0	128	64 × 64 × 23
Leiden	Rombouts, S.A.R.B.	12	2.2	215	64 × 64 × 38
Leiden	Rombouts, S.A.R.B.	19	2.2	215	64 × 64 × 38
Leipzig	Villringer, A.	37	2.3	195	64 × 64 × 34
Milwaukee	Li, S.J.	18	2.0	175	64 × 64 × 20
Milwaukee	Li, S.J.	46	2.0	175	64 × 64 × 36
Munchen	Sorg, C., Riedl, V.	16	3.0	72	64 × 64 × 33
Newark	Biswal, B.	19	2.0	135	64 × 64 × 32
New Haven	Hampson, M.	19	1.0	249	64 × 64 × 16
New Haven	Hampson, M.	16	1.5	181	64 × 64 × 22
New York	Milham, M.P., Castellanos, F.X.	25	2.0	192	64 × 64 × 39
New York	Milham, M.P., Castellanos, F.X.	84	2.0	192	64 × 64 × 39
New York	Milham, M.P., Castellanos, F.X.	20	2.0	175	64 × 80 × 33
Orangeburg	Hoptman, M.	20	2.0	165	64 × 64 × 22
Oulu	Kiviniemi, V.J., Veijiola, J.	103	1.8	245	64 × 64 × 28
Oxford	Smith, S.M., Mackay, C.	22	2.0	175	64 × 64 × 34
Palo Alto	Greicius, M.	17	2.0	235	64 × 64 × 29
Pittsburgh	Siegle, G.	17	1.5	275	64 × 64 × 29
Queensland	McMahon, K.	19	2.1	190	64 × 64 × 36
Saint Louis	Schlaggar, B., Petersen, S.	31	2.5	127	64 × 64 × 32
Taipei	Lin, C.P.	14	2.0	295	64 × 64 × 32
Taipei	Lin, C.P.	8	2.0	175	64 × 64 × 33

The New Haven data comprised two or four resting-state data sets per subject; the ICBM data provided three data sets per subject. This yields a total of 1484 resting-state data sets (parts of the original table in Eklund et al., 2012).

Eklund et al. analyzed the data sets using SPM8 with different options. They always applied motion correction, high-pass filtering, and the implemented prewhitening procedure using the AR(1) correlation model. Their analysis was run both with (variant A below) and without (variant B) global normalization and the use of additional regressors for motion correction in the design matrix. Furthermore, the authors analyzed seven degrees of smoothing (4, 6, 8, 10, 12, 14, and 16 mm Gaussian kernel) as well as eight simulated designs: four block-based designs (alternating activity and rest periods of 10, 15, 20, and 30 s, respectively), and four event-related designs (periods of 1 up to 8 s, partially randomized). The stimulus paradigms, convolved with the canonical hemodynamic response function, were used together with their derivatives as regressors. They then always applied the FWE adjustment for multiple testing assuming a 5% level of significance.

Additionally, the authors compared the SPM8 results for variant B with a nonparametric approach applying a random permutation test with 10,000 permutations to each dataset. Prior to this, high-pass filtering and prewhitening with a voxelwise AR(4) model were applied to ensure the exchangeability of the samples under the null hypothesis. Due to the long computational time, only one smoothing procedure with a Gaussian kernel of FWHM 8 mm was applied in the permutations.

Because the resting-state data should have no correlation to the simulated artificial designs, the nominal level of significance should be ensured by using FWE correction, i.e., no activated voxel should be detected in about 95% of the data sets analyzed. Taking into account the random nature of this rate, Eklund et al. should have observed a rate of data sets with at least one significant voxel among all 1482 sets that is covered by the interval [3.9%; 6.1%] [95% confidence interval; cf. Eklund et al. (2012)]. In fact, the observed rates were much larger. A summary of selected results from Eklund et al. is presented in Table 2. Here we focus on the block-based paradigm of activity and rest periods of 30 s each, which was also used in our re-analyses with block permutation. The empirical familywise error rates in Eklund et al. were far above the nominal test level for all analyzed degrees of smoothing as well as for the two model variants A and B (including or excluding global normalization and motion regressors, respectively).

**Table 2:** **Results (rate of false positive findings) of Eklund et al. (2012) using a block-based design with activity and rest periods of 30 s each**.

**Variant**	**Smoothing (mm)**	**FWE**	**Stratified according to TR**		
			**FWE *TR* = 1 s**	**FWE *TR* = 2 s**	**FWE *TR* = 3 s**
A	4	0.438	0.722	0.439	0.182
	6	0.375	0.629	0.377	0.147
	8	0.343	0.557	0.348	0.136
	10	0.342	0.546	0.334	0.147
	12	0.321	0.474	0.314	0.141
	14	0.311	0.443	0.303	0.136
	16	0.294	0.464	0.290	0.106
B	4	0.375	0.670	0.381	0.192
	6	0.315	0.577	0.318	0.150
	8	0.274	0.536	0.271	0.122
	10	0.257	0.505	0.258	0.126
	12	0.248	0.485	0.251	0.108
	14	0.240	0.454	0.243	0.112
	16	0.215	0.412	0.227	0.084
	8 (Permutation)	0.075	0.124	0.089	0.056
95% CI		[0.039; 0.061]	[0.007; 0.093]	[0.035; 0.065]	[0.021; 0.079]
Number of datasets		*n* = 1482	*n* = 97	*n* = 796	*n* = 214

Data were spatially smoothed. A: including global normalization and motion regressors, B: excluding global normalization and motion regressors. The table summarizes the supplementary material from their paper presented at http://people.imt.liu.se/andek/rest_fMRI/ (accessed on 24.04.2013).

The rates varied according to the type of design (block- or event-related), the design parameters, smoothing, inclusion of normalization and motion regressors in the model, and, particularly, the repetition times. Hence, we also use the stratification with respect to repetition time for the results of our blockwise permutation approach listed below.

The results for the permutation tests after prewhitening with a voxelwise AR(4) model (“Permutation” row in Table 2) were closer to the expected rate of 0.05. But only for a long TR of 3 s the observed FWE was included in the 95% confidence interval for the design considered here.

### 2.2. The new concept of a blockwise permutation including a random shift

In contrast to an AR(1) model's strict assumption, our proposal (Adolf et al., 2011) merely assumes that the correlation of subsequent measurements decreases according to their temporal distance. This more general and less constraining assumption includes but is not restricted to a AR(1) process. However, also this method requires temporal stationarity of the noise process.

For the ease of the reader we briefly review our approach. The time series is split into blocks of adjacent elements. We then consider these blocks as permutation elements and permute them as a whole. An approximate exchangeability of these blocks under the null hypothesis can be achieved by a sufficiently large block length *l* given the correlation structure described above. The block length *l* can be chosen within 1 ≤ *l* ≤ *n*/2, where *n* is the number of data points in the time series. It directly affects the remaining correlation of blocks. For example, given an AR(1) autocorrelation structure with a correlation coefficient of ρ = 0.4, the geometric mean of the pairwise correlation coefficients of two neighboring blocks is ρ^*l*^, which is lower than 0.001 with a block length of *l* = 10. Thus, it would be sufficient here to define a block length of ten adjacent elements yielding approximately exchangeable blocks under the null hypothesis.

Because this procedure implies that the number of possible permutations is dependent on the number of blocks, there must still be enough blocks to yield a sufficiently detailed permutation distribution. To increase the number of possible permutations, we use a random shift in each permutation step. This means that a random number of elements is removed from the beginning of the time series and added to the end before splitting it into blocks. Here it is advantageous if the block length *l* is no divisor of the time series length *n*, *n mod l* = *r* (*r* > 0). Then, the last block has a length of (*l* + *r*) and thus differs from the other ones with length *l*. This procedure is similar to a moving bootstrap approach (Politis and Romano, 1991).

Additionally, this random shift procedure ensures that the blockwise splitting of the time series is not unintentionally synchronous to the paradigm, which is a prerequisite for applying the method to block-based fMRI designs.

As with many other software packages, we do not systematically execute all possible permutations but use instead a set of random permutations.

The permutation is not carried out in the vector *y* of the observed BOLD signals of a given voxel but in the hypothesis-related part of the design matrix. That way, a potentially significant effect can truly be attributed to the tested parameters. Additionally this procedure ensures automatically that the permutations are identical over all voxels considered simultaneously which is necessary for the Westfall-Young procedure considered below.

In order to separate the contrasts between different conditions from the rest of the parameter space, the general linear model is transformed via an orthogonal decomposition into a model with two orthogonal parts concerning the design matrix and the corresponding parts of the parameter vector:

$$y=X_{1}\beta_{1}\;+\;X_{2}\beta_{2}\;+\;\epsilon ,$$

where *X*_2_ is the hypothesis-related part of the design (the contrast(s) of interest) and *X*_1_ corresponds to the remaining parameters whose potentially confounding effects need to be eliminated. Thus, the corresponding null hypothesis is H_0_: β_2_ = 0.

To demonstrate the validity of our approach with respect to the type I error rate and the dependence on the block length, a small simulation study was performed. The chosen design resembles a block-based paradigm with 20 runs of activation and rest periods, respectively, resulting in a total number of 420 scans. Time series for a total of 500 voxels were simulated under the null hypothesis based on normal distributed data with implemented temporal and spatial dependencies. The temporal correlation was modeled assuming an AR(1) process with a correlation coefficient of ρ = 0.4. For the simulation of spatial dependence we used a block compound symmetry structure with three functional groups of voxels that are mutually independent but within each group are all correlated with the same pairwise correlation coefficient of 0.5. A univariate *t*-test statistic was determined for each of the 500 variables within the permutation approach. The adjustment for multiple testing was carried out according to the Westfall-Young principle, where in each permutation step the successive maxima of permuted test statistics per voxel were determined and compared to originally ranked ones. The adjusted *p*-values were thereby derived analogous to the maxT procedure (step-down procedure) described in Westfall and Young (1993). We used 299 random permutations and a total number of 2500 repetitions. Given this number of replications and a nominal error level of 0.05, the empirical familywise error rate should be approximately covered by the interval [0.0415; 0.0585] with probability 0.95. The simulations were repeated with different block lengths from *l* = 1, which is a classical elementwise permutation, to *l* = 60.

### 2.3. Analysis of the resting-state data

For our analysis of the resting-state data we used the same data sets as considered in Eklund et al. (2012), excluding 19 data sets: 16 data sets from Sorg and Ridel had only 72 time points, which would have overly limited the number of blocks in the permutation procedure, two data sets could not be used because they had empty brain masks (and had already been excluded by Eklund et al.), and one could not be opened. Thus, 1465 data sets were used for our re-analyses.

We applied the same preprocessing, simulated paradigms, and regressors in the model as used by Eklund et al. apart from the following restriction: given the long computational time required by the permutation tests, we considered only the simulated block-designs with activation and rest periods of 30 s each as these designs yielded the highest false-positive rates of all designs considered in Eklund et al. (2012). Furthermore, we used only the 8 mm Gaussian kernel for spatial smoothing that had been applied in both the parametric and permutation approach of (Eklund et al.).

To apply our concept of a blockwise permutation including a random shift on the resting-state data sets and to make our analyses as comparable as possible to those of Eklund et al., we included SPM8 components in the MATLAB code for the permutation procedure and combined both into a batch mode. To shorten computation time we used MATLAB 7.11.0 (R2010b), including MATLAB's Parallel Computing Toolbox on an Intel(R) Core(TM) i5 with 4 GB of RAM.

Functions of SPM8 (Revision Number 4667) were used for creating the design and applying the canonical hemodynamic response function and its temporal derivative. In line with Eklund's analyses, motion regressors and global normalization were included in variant A but not in variant B. Because the temporal correlation is addressed in the block permutation principle in our approach, the AR(1) autocorrelation correction was disabled in both variants. Thus the SPM8 model estimation was performed without prewhitening. Only high-pass filtering was included.

The resulting *t*-values from the original (not permuted) data were compared with the corresponding results of 999 repetitions after applying random blockwise permutations including a random shift in the design matrix, as described in Section 2.2. To simplify the computational implementation and to maintain comparability of the results among themselves, we chose a uniform block length of 23 adjacent elements for analyzing all data sets. Because 23 is not a divisor of any of the sample sizes of the analyzed data sets, the number of possible blockwise permutations is enlarged by this strategy to *k*!· *n*, with *k* and *n* being the number of blocks and the sample size, respectively (for details, see Adolf et al., 2011). A block length of 23 should be large enough to yield approximately exchangeable blocks under the null hypothesis. Furthermore, this length was small enough to ensure a sufficiently detailed resolution of the permutation distribution in the included 1465 resting-state data sets. Variants A and B were performed separately.

The SPM model estimation is repeated in each permutation step. The so-called maxT procedure of Westfall and Young (1993) was used to control the familywise type I error for the simultaneous analysis of all voxels: within one permutation step (*i*_*perm*_ = 1, …, *n*_*perm*_) the *t*-values *t*_*i*_*perm*__ are sorted and the maximum *t*-value is compared to the maximum *t*-value of the original model estimation *t*_0_. Given that we were interested only in testing whether at least one voxel is significant, we omitted the further stepdown steps from the original procedure and thus reduced the computational load. Ultimately, the adjusted *p*-value is given by

$$p=\frac{N_{1}+1}{n_{perm}+1},$$

where *N*_1_ is the number of permutations fulfilling the condition *max* (|*t*_*i*_*perm*__|) ≥ *max* (|*t*_0_|) and *n*_*perm*_ is the number of random permutations. If this *p*-value is less than or equal to the 5% significance level, the corresponding data set is marked as false positive. Finally, the rate of false-positive data sets is computed.

The blockwise permutation approach is implemented in an SPM toolbox “StabMultip” developed by our work group. For multiple adjustment the toolbox provides the Westfall-Young method as well as a class of FWE-controlling multiple test procedures based on data-driven ordered or weighted hypotheses. The toolbox “StabMultip” can be downloaded from the website http://www.med.uni-magdeburg.de/fme/institute/ibmi/download/StabMultip.zip.

## 3. Results

The results of the simulation series (cf. Section 2.2) are displayed in Table 3. Shown are the empirical familywise type I errors of the blockwise permutation with random shift according to the block length. The rejection rate of 0.810 in the classical elementwise permutation (block length = 1) indicates the strong violation of the nominal FWE. This violation decreases with increasing block length, so that for the assumed moderate temporal AR(1) correlation of 0.4 in this series an approximate error control is given for a block length of at least 20. The required block length to approximately maintain the nominal test level also depends on the underlying test statistic. More extended simulations with an emphasis on multivariate test statistics are described in Adolf et al. (2011). There, it was shown that larger block lengths (up to about 40) may be necessary in multivariate test scenarios.

**Table 3:** **Empirical familywise type I error of the blockwise permutation including a random shift with an underlying Westfall-Young test procedure for different block lengths (temporal AR(1) correlation ρ = 0.4, *n* = 420, *p* = 500, α = 0.05, 299 random permutations, 2500 replications)**.

**Block length**	**1**	**5**	**10**	**15**	**20**	**25**	**40**	**50**	**60**
*p*− value	0.810	0.113	0.071	0.068	0.054	0.054	0.049	0.049	0.050

**Figure 1:**
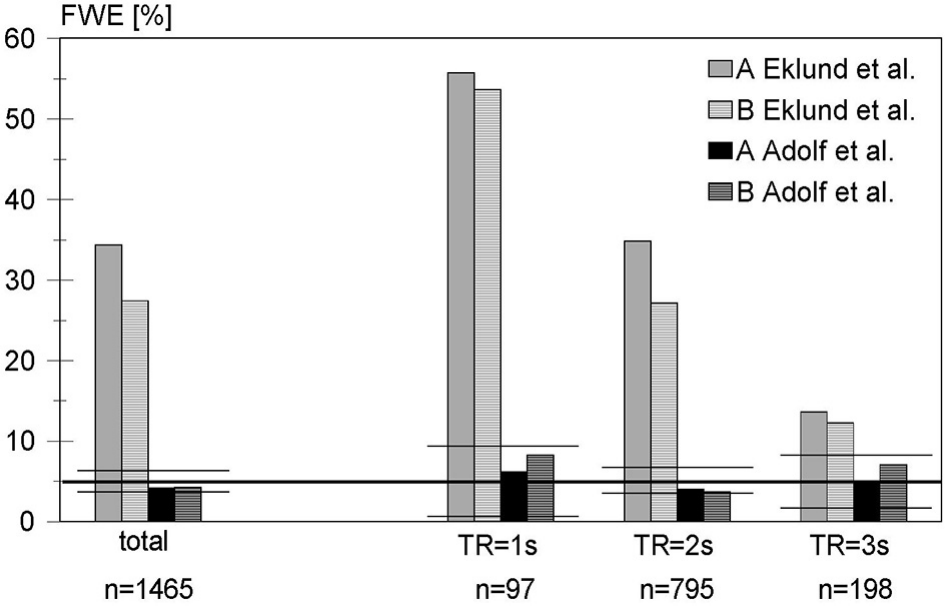
**False-positive rates of the blockwise permutation method for analyzing 1465 resting-state data sets using a block-based design with activity and rest periods of 30 s each, compared with the corresponding results from Eklund et al**. A: including global normalization and use of motion regressors in linear model, B: excluding global normalization and motion regressors (see text for further details). Because the repetition time is an important factor in the discussion of Eklund's results, our results were stratified accordingly. To facilitate the comparison with Eklund, the confidence intervals are marked by horizontal horizontal lines.

In the next step, real resting-state data were analyzed. Figure 1 shows the empirical familywise error rates over all data sets considered with our blockwise permutation procedure as compared with those from Eklund et al. (2012). Based on the variance formula of the binomial distribution, the percentage of data sets with at least one significant voxel of all 1465 resting-state data sets, i.e., the empirical FWE should be covered by the interval [0.0388; 0.0612] with probability 0.95. In fact, using the blockwise permutation including a random shift, the FWE is 0.0416 in the analysis including a global normalization and motion regressors (variant A) and 0.0423 in variant B excluding both.

Stratifying the results according to the repetition time revealed that all false-positive rates in our analysis were within the corresponding confidence intervals. Even for short TR, which implies a higher autocorrelation between successive scans and thus may result in higher false-positive rates, the nominal familywise test level was approximately maintained. Thus, detected false-positive rates were much lower than with the standard approach of using SPM, as used by Eklund et al. For TR = 1 s the FWE was 0.0619 (variant A) and 0.0825 (variant B), which is still below the 95% upper confidence limit of 0.0934. For TR = 2 s the FWE was 0.0403 (variant A) and 0.0365 (variant B) and therefore within the interval [0.0348; 0.0652]. For a TR = 3 s (only the data sets of one large study, cf. Table 1), the FWE was 0.0505 (variant A) and 0.0707 (variant B) with a 95% confidence interval of [0.0196; 0.0804].

**Figure 2:**
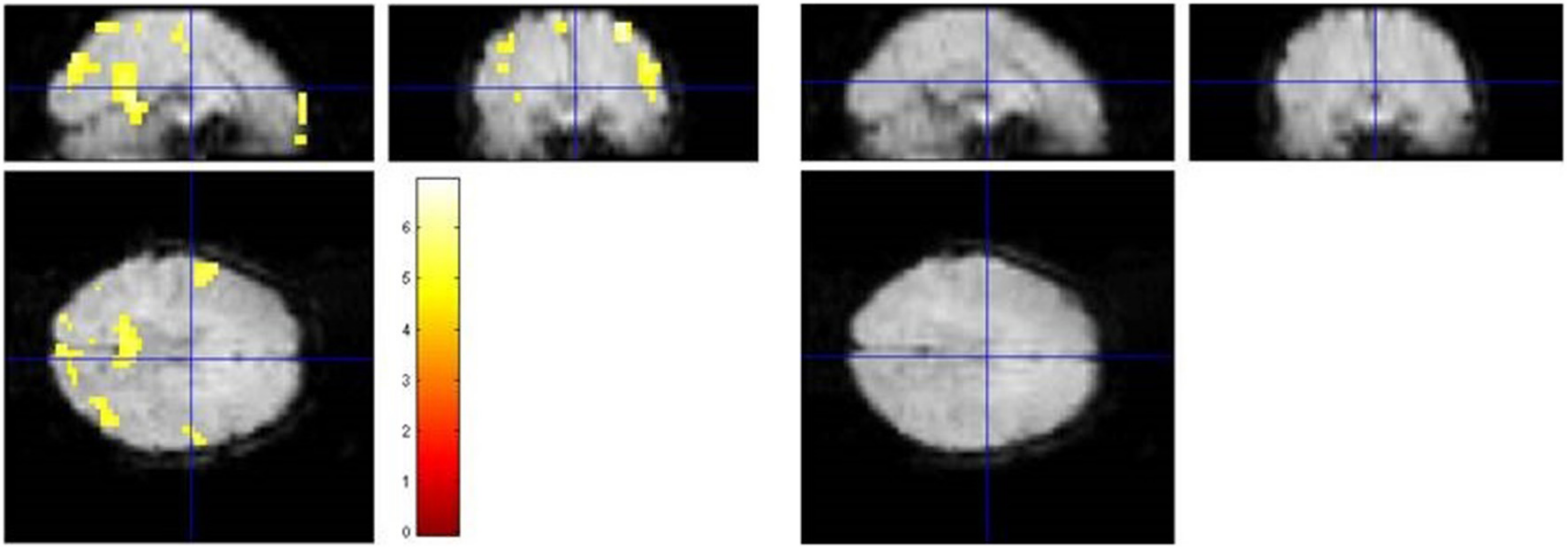
**Illustration of the results of a standard SPM analysis and the proposed blockwise permutation model of a representative subject (No. 1007 of the resting-state data used in Eklund et al., 2012). Left:** The SPM analysis with AR(1) model assuming a block design yielded 666 activated voxels (*p* < 0.05, FWE corrected) in different brain regions. **Right:** Applying the blockwise permutation method reduced the number of voxels, leaving no significant voxel at all in this case.

For illustration. Figure 2 shows the spatially resolved results of one representative data set (#1007 from Eklund et al.). Clearly, the false-positive results of the parametric GLM analysis including an AR(1) model were no longer detectable in the blockwise permutation method.

## 4. Discussion

The exemplary analyses of resting-state data sets presented here show that our nonparametric approach yields valid results in fMRI analyses. The nominal familywise test level can be approximately maintained in all simulations performed. Of course, this is not a definitive proof that our approach will lead to the same improvements in all fMRI data analyses. However, the approach successfully and strongly decreased the number of apparently activated voxels in those designs that in Eklund et al. (2012) showed the largest violations of the nominal FWE when applying standard SPM analyses.

Eklund et al. also performed logistic regression analyses of the influence of different aspects—namely block design vs. event-related design, repetition time, preprocessing procedures (smoothing, normalization), and inclusion of motion regressors in the linear model—on the rejection rates in the classical parametric approach based on the AR(1) assumption. They found dependencies of the FWE control on all the above factors, including two- and three-way interactions. Thus, the interdependencies between these factors are intricate and in general there are no simple empirically based adjustment factors for the nominal familywise error rate for a specific situation.

In addition, Eklund et al. extensively investigated the spectra of the original and prewhitened residuals in the classical parametric approach. This led them to the final conclusion that the global AR(1) autocorrelation correction in SPM failed to model the spectra of the residuals, which seemed to be the main reason for these high familywise error rates.

There are several alternative methods that take into account the temporal autocorrelation. The most common one is a Satterthwaite correction of the variance estimation, as described in Veltman and Hutton (2001) or Frackowiak et al. (2004). Bullmore et al. (2001) and Friman and Westin (2005) proposed a correction in the wavelet domain instead of the time domain. In order to reduce the dependency of estimation and correction methods on specific model assumptions, Friston et al. (2000) recommended the inclusion of smoothing procedures.

Although the use of permutation techniques (Nichols and Holmes, 2001) generally reduces model dependency in statistical tests, temporal correlation remains an issue because it undermines the distributional exchangeability of the sample elements, which is the crucial assumption in permutation tests. Therefore, permutation is usually combined with prewhitening (Bullmore et al., 2001; Frackowiak et al., 2004, chapter 46) at the price of again being dependent on the model assumptions about temporal correlation. This can be seen in permutation test results in Eklund et al. (2012). Although they used a more general AR(4)-model for prewhitening and succeeded in reducing the violations of the familywise error rates, the rejection rates were still above the nominal FWE in some of the considered configurations. The influence of the repetition time is not quite consistent over the different experimental designs in Eklund's paper (not all included in the present paper). That might be the result of two contrary effects. On the one hand larger repetition times decrease the serial correlation and thus the dependency on the exact model, on the other hand they are usually associated with a smaller number of repetitions that complicates the estimation of the model parameters.

Our block permutation approach avoids this prewhitening step and thus rigorously overcomes the restriction to a special model like AR(1). That leads to the more reliable analyses observed with respect to controlling the type I error. In order to take into account the other confounding factors, we permuted only the column(s) of the design matrix that are associated with the null hypothesis of the test. Similar approaches were used in Hummel et al. (2008) to analyze independent gene expression data, and have already been described in Nichols and Holmes (2001) paper on applying permutation tests in neuroimaging in order to eliminate the confounding effect of the covariable global cerebral blood flow on a PET signal. This approach approximately corresponds to the permutation of residuals (ter Braak, 1992).

In our investigations we did not observe negative effects of block resampling as reported by Davison and Hinkley (1997) or Bullmore et al. (2001). They reported that the block bootstrapped series might be “whiter” than the original series with possibly strong consequences for further inference which led to the development of special bootstrap procedures by Carlstein et al. (1998). One reason that we did not observe this could be that we used permutation instead bootstrap (with replacement), large block sizes and the random shift. But possibly the main difference to the counter-example of Davison is that our goal is not the estimation of the serial correlation itself. Instead we want to test the influence of a regressor in the linear model where the serial correlation is only a technical complication which has to be taken into account. The described method to permute the corresponding column of the design matrix instead the observations has the advantage that the observed time series and the other possibly influencing regressors are not changed during permutation, only that regressor which has no influence under the null hypothesis. It should be noted here that we considered only voxel level inference here and no cluster level inference. Eklund et al. (2012) had also considered cluster level inference in their permutation approach and found a significant influence of the inference level on the type I error control.

One might argue that the improved type I error control is associated with a lowered power under true alternative hypotheses. But the results of multivariate analyses with real and simulated data in Adolf et al. (2011) showed that the blockwise permutation including a random shift is also a very powerful tool. Particularly with real data, this nonparametric method, which is based on less restrictive model assumptions, yielded more significant results than the parametric approaches. Thus, our approach ensures powerful results without compromising control of the FWE. But even if there were some loss of power in specific situations, maintaining the type I error should always have priority.

A crucial step in the blockwise permutation method is the choice of the block length. As long as the number of possible permutations is large enough, small block lengths should be avoided. Based on our experience from different simulation studies, we recommend a block length of at least 20 measurements in univariate analyses at the voxel level. Otherwise the potential for false-positive results increases. However, the number of possible permutations must remain large enough to rule out large jumps in the permutation distribution and thus to allow sufficiently small *p*-values. This issue is already discussed in the monograph of Davison (1997, chapter 8.3). Including the random shift in the block permutation reduces this problem considerably. Nevertheless, we recommend choosing a block length that yields at least four or five blocks. If one has doubts about the choice of the permutation setting in a given real data set, then it could be helpful to repeat the analysis many times with randomly chosen artificial paradigms that should be sufficiently independent from the real paradigm. Then the rejection rate over the repetitions should be near the nominal α-level (similar as in the evaluation of the resting state data in the present paper).

In this context, it is important to note our choice of the Westfall-Young procedure for controlling the FWE familywise error instead of the Bonferroni procedure or modifications of it. By using the Westfall-Young procedure, we avoid the small adjusted error levels for the voxelwise analyses and carry out all analyses at the nominal familywise level. Such smaller error levels would require not only a larger number of (theoretically) possible permutations but also a correspondingly large number of random permutations. That would increase the computing time.

Obviously, the permutation approach involves some computational load. In the presented Westfall-Young version it took on the average about three hours to perform a test with 999 random permutations for one data set on our computer. Therefore, the method provides an alternative for offline analyses only. In the case of these resting-state data sets it would have taken months to repeat all of Eklund et al. (2012) analyses using the blockwise permutation method, which forced us to restrict the analyses.

## 5. Conclusions

Our re-assessment of 1465 sets of resting-state fMRI data for artificially assumed paradigms analyzed by Eklund et al. (2012), in which we applied our new approach of blockwise permutation including a random shift, led to much better control of the familywise error rates. The method therefore provides a promising tool for increasing the quality and reliability of fMRI data analyses.

### Conflict of interest statement

The authors declare that the research was conducted in the absence of any commercial or financial relationships that could be construed as a potential conflict of interest.

## References

[B1] Adolf D. Baecke S. Kahle W. Bernarding J. Kropf S. (2011). Applying multivariate techniques to high-dimensional temporally correlated fMRI data. J. Stat. Plan. Infer. 141, 3760–3770 10.1016/j.jspi.2011.06.012

[B2] Ahmadi A. Pearlson G. Meda S. Dager A. Potenza M. Rosen R. (2013). Influence of alcohol use on neural response to go/no-go task in college drinkers. Neuropsychopharmacology 38, 2197–2208 10.1038/npp.2013.119 23670589 PMC3773670

[B3] Ashburner J. Barnes G. Chen C. Daunizeau J. Flandin G. Friston K. (2013). SPM8 Manual. London: Wellcome Trust Centre for Neuroimaging

[B4] Baecke S. Luetzkendorf R. Tempelmann C. Mueller C. Adolf D. Scholz M. (2009). Event-related functional magnetic resonance imaging (efMRI) of depth-by-disparity perception: additional evidence for right-hemispheric lateralization. Exp. Brain Res. 196, 453–458 10.1007/s00221-009-1844-z 19471910

[B5] Benjamini Y. Hochberg Y. (1995). Controlling the false discovery rate: a practical and powerful approach to multiple testing. J. R. Stat. Soc. 57, 289–300

[B6] Biswal B. Mennes M. Zuo X. Gohel S. Kelly C. Smith S. (2010). Toward discovery science of human brain function. Proc. Natl. Acad. Sci. U.S.A. 107, 4734–4739 10.1073/pnas.0911855107 20176931 PMC2842060

[B7] Bullmore E. Long C. Suckling J. Fadili J. Calvert G. Zelaya F. (2001). Coloured noise and computational inference in neurophysiological (fMRI) time series analysis: resampling methods in time and wavelet domains. Hum. Brain Mapp. 12, 61–78 10.1002/1097-0193(200102)12:2<61::AID-HBM1004>3.0.CO;2-W 11169871 PMC6871881

[B8] Carlstein E. Do K. Hall P. Hestenberg T. Künsch H. (1998). Matched-block bootstrap for dependent data. Bernoulli 4, 305–328 10.2307/3318719

[B9] Causse M. Peran P. Dehais F. Caravasso C. Zeffiro T. Sabatini U. (2013). Affective decision making under uncertainty during a plausible aviation task: An fMRI study. Neuroimage 71, 19–29 10.1016/j.neuroimage.2012.12.060 23313780

[B10] Davison A. Hinkley D. (1997). Bootstrap Methods and Their Application. Cambridge: Cambridge University press 10.1017/CBO9780511802843

[B11] Di X. Kim E. Huang C. Tsai S. Lin C. Biswal B. (2013). The influence of the amplitude of low-frequency fluctuations on resting-state functional connectivity. Front. Hum. Neurosci. 7:118 10.3389/fnhum.2013.00118 23565090 PMC3613753

[B12] Eklund A. Andersson M. Josephson C. Johannesson M. Knutsson H. (2012). Does parametric fMRI analysis with SPM yield valid results? - An empirical study of 1484 rest datasets. Neuroimage 61, 565–578 10.1016/j.neuroimage.2012.03.093 22507229

[B13] Fair D. Cohen A. Power J. Dosenbach N. Church J. Miezin F. (2009). Functional brain networks develop from a “local to distributed” organization. PLoS Comput. Biol. 5:e1000381 10.1371/journal.pcbi.1000381 19412534 PMC2671306

[B14] Focke N. Yogarajah M. Bonelli S. Bartlett P. Symms M. Duncan J. (2008). Voxel-based diffusion tensor imaging in patients with mesial temporal lobe epilepsy and hippocampal sclerosis. Neuroimage 40, 728–737 10.1016/j.neuroimage.2007.12.031 18261930

[B15] Frackowiak R. Friston K. Frith C. Dolan R. Price C. Zeki S. (2004). Human Brain Function, 2 Edn, London: Elsevier/Academic Press

[B16] Friman O. Westin C. (2005). Resampling fMRI time series. Neuroimage 25, 859–867 10.1016/j.neuroimage.2004.11.046 15808986

[B17] Friston K. Ashburner J. Kiebel S. Nichols T. Penny W. (2007). Statistical Parametric Mapping - The Analysis of Functional Brain Images. San Diego, CA: Elsevier/Academic Press

[B18] Friston K. Josephs O. Zarahn E. Holmes A. Rouquette S. Poline J. (2000). To smooth or not to smooth? bias and efficiency in fMRI time-series analysis. Neuroimage 12, 196–208 10.1006/nimg.2000.0609 10913325

[B19] Genovese C. Lazar N. Nichols T. (2002). Thresholding of statistical map in functional neuroimaging using the false discovery rate. Neuroimage 15, 870–878 10.1006/nimg.2001.1037 11906227

[B20] Groeschel S. Sohns J. Schmidt-Samoa C. Baudewig J. Becker L. Dechent P. (2013). Effects of age on negative BOLD signal changes in the primary somatosensory cortex. Neuroimage 71, 10–18 10.1016/j.neuroimage.2012.12.039 23296182

[B21] Hummel M. Meister R. Mansmann U. (2008). Globalancova: exploration and assessment of gene group effects. Bioinformatics 24, 78–85 10.1093/bioinformatics/btm531 18024976

[B22] Kahnt T. Tobler P. (2013). Salience signals in the right temporoparietal junction facilitate value-based decisions. J. Neurosci. 33, 863–869 10.1523/JNEUROSCI.3531-12.2013 23325225 PMC6704859

[B23] Meda S. Calhoun V. Astur R. Turner B. Ruopp K. Pearlson G. (2009). Alcohol dose effects on brain circuits during simulated driving: an fmri study. Hum. Brain Mapp. 30, 1257–1270 10.1002/hbm.20591 18571794 PMC2751645

[B24] Morcom A. Friston K. (2012). Decoding episodic memory in ageing: a bayesian analysis of activity patterns predicting memory. Neuroimage 59, 1772–1782 10.1016/j.neuroimage.2011.08.071 21907810 PMC3236995

[B25] Nichols T. Holmes A. (2001). Nonparametric permutation tests for functional neuroimaging: a primer with examples. Hum. Brain Mapp. 15, 1–25 10.1002/hbm.1058 11747097 PMC6871862

[B26] Politis D. Romano J. (1991). A circular block-resampling procedure for stationary data. Technical Report No. 91-07. Lafayette, LA: Department of Statistics, Purdue University

[B27] Scarpazza C. Sartori G. Simone M. D. Mechelli A. (2013). When the single matters more than the group: very high false positive rates in single case voxel based morphometry. Neuroimage 70, 175–188 10.1016/j.neuroimage.2012.12.045 23291189

[B28] Silver M. Montana G. Nichols T. (2011). Alzheimer's disease neuroimaging initiative, false positives in neuroimaging genetics using voxel-based morphometry data. Neuroimage 54, 992–1000 10.1016/j.neuroimage.2010.08.049 20849959 PMC3063336

[B29] Smith A. Lewis B. Ruttimann U. Ye F. Sinnwell T. Yang Y. (1999). Investigation of low frequency drift in fmri signal. Neuroimage 9, 526–533 10.1006/nimg.1999.0435 10329292

[B30] Sorg C. Riedl V. Mühlau M. Calhoun V. Eichele T. Läer L. (2007). Selective changes of resting-state networks in individuals at risk for alzheimer's disease. Proc. Natl. Acad. Sci. U.S.A. 104, 18760–18765 10.1073/pnas.0708803104 18003904 PMC2141850

[B31] ter Braak C. (1992). Permutation versus bootstrap significance tests in multiple regression and ANOVA, in Bootstrapping and Related Techniques, ed Jockel K. J. (Berlin: Springer-Verlag), 79–85

[B32] Veltman D. Hutton C. (2001). SPM99 Manual. London: Wellcome Trust Centre for Neuroimaging

[B33] Westfall P. Young S. (1993). Resampling-Based Multiple Testing: Examples and Methods for p-value Adjustment. New York, NY: John Wiley & Sons

